# UO_2_ dissolution in bicarbonate solution with H_2_O_2_: the effect of temperature†

**DOI:** 10.1039/d2ra08131h

**Published:** 2023-09-22

**Authors:** John McGrady, Yuta Kumagai, Yoshihiro Kitatsuji, Akira Kirishima, Daisuke Akiyama, Masayuki Watanabe

**Affiliations:** a Nuclear Science and Engineering Center, Japan Atomic Energy Agency (JAEA) Tokai Ibaraki 319-1195 Japan j.mcgrady@kyotofusioneering.com kumagai.yuta@jaea.go.jp; b Institute of Multidisciplinary Research for Advanced Materials, Tohoku University 1-1 Katahira, 2-chome, Aoba-ku Sendai 980-8577 Japan

## Abstract

Upon nuclear waste canister failure and contact of spent nuclear fuel with groundwater, the UO_2_ matrix of spent fuel will interact with oxidants in the groundwater generated by water radiolysis. Bicarbonate (HCO_3_^−^) is often found in groundwater, and the H_2_O_2_ induced oxidative dissolution of UO_2_ in bicarbonate solution has previously been studied under various conditions. Temperatures in the repository at the time of canister failure will differ depending on the location, yet the effect of temperature on oxidative dissolution is unknown. To investigate, the decomposition rate of H_2_O_2_ at the UO_2_ surface and dissolution of U^VI^ in bicarbonate solution (0.1, 1, 10 and 50 mM) was analysed at various temperatures (10, 25, 45 and 60 °C). At [HCO_3_^−^] ≥ 1 mM, the concentration of dissolved U^VI^ decreased with increasing temperature. This was attributed to the formation of U^VI^-bicarbonate species at the surface and a change in the mechanism of H_2_O_2_ decomposition from oxidative to catalytic. At 0.1 mM, no obvious correlation between temperature and U dissolution was observed, and thermodynamic calculations indicated this was due to a change in the surface species. A pathway to explain the observed dissolution behaviour of UO_2_ in bicarbonate solution as a function of temperature was proposed.

## Abstract 2

U dissolution *via* H_2_O_2_ decomposition on UO_2_ powder in NaHCO_3_ solution decreased with increasing temperature. This was caused by a transition from oxidative to catalytic decomposition with temperature.

## Graphical abstract

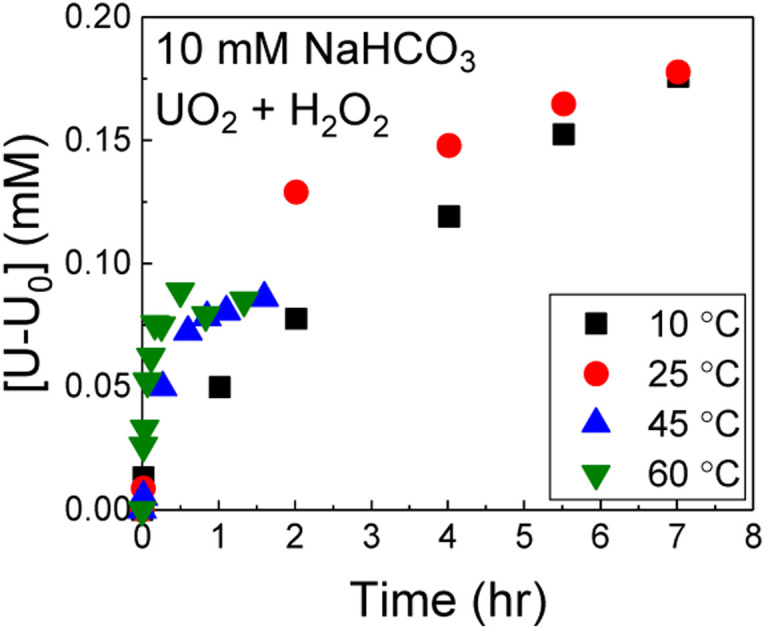

## Introduction

Ensuring the safe disposal of spent nuclear fuel provides numerous engineering and technological challenges to the global nuclear community. A current potential strategy for spent fuel disposal is the use of deep geological repositories which provide a long-term solution for spent fuel storage needs. The repository barriers between spent fuel and the local environment have been designed to endure, yet inevitably these barriers will breakdown leading to the release of radioactive species from spent fuel. The dominant mechanism of radionuclide release in such an event is predicted to be due to the interaction of groundwater with the spent fuel surface, leading to dissolution and subsequent transport of the radionuclides from the repository to the environment.

UO_2_ is the main constituent of spent fuel making up around 95%, while the remaining 5% consists of fission products and heavier actinide species. Typical groundwater at repository depths is reducing and anoxic, and the solubility of U^IV^ under such conditions is very low. Therefore, significant dissolution of U from UO_2_ to groundwater may not be expected. However, ionizing radiation from the spent fuel will cause radiolysis of the groundwater, and oxidising radiolysis products (such as H_2_O_2_, O_2_, OH˙) will be generated at the spent fuel surface. This will have a significant effect on the redox chemistry at the surface and the rate of UO_2_ oxidation. As the solubility of U^VI^ is significantly higher than U^IV^ in typical groundwater conditions,^1,2^ oxidation of the surface is expected to have a large impact on U dissolution.

Studies have identified H_2_O_2_ as the primary radiolysis product of concern with regards to UO_2_ oxidation,^3,4^ and two pathways for the reaction of H_2_O_2_ with UO_2_ have been proposed.^5,6^ Catalytic decomposition of H_2_O_2_ involves adsorption of H_2_O_2_ onto the UO_2_ surface, followed by surface-catalyzed splitting of the O–O peroxide bond according to reactions (1)–(3), whilst the second decomposition pathway is *via* an oxidative decomposition mechanism according to reaction (4):

1 UO_2_ + 1/2H_2_O_2_ → UO_2_ − (˙OH)_ads_

2 UO_2_ − (˙OH)_ads_ + H_2_O_2_ → UO_2_ + HO_2_ + H_2_O

3 2(HO_2_˙) → H_2_O_2_ + O_2_

4 UO_2_ − (˙OH)_ads_ → UO_2_^+^ + OH^−^

Typically, bicarbonate (HCO_3_^−^) is found in groundwater at various concentrations depending on repository location (∼10^−4^ to ∼10^−2^ M).^7–10^ Bicarbonate forms complexes with oxidised U and promotes dissolution *via* stabilisation of the dissolution products:

5 U^(VI)^(CO_3_)_ads_ + HCO_3_^−^ → (U^(VI)^O_2_(CO_3_)_2_)^2−^ + H^+^

Due to the importance of understanding U dissolution for the development of predictive models for radionuclide release, previous studies have investigated the dissolution of U under simulated groundwater conditions. Such studies on the oxidative dissolution of U include the effects of: the form of U,^11,12^ the radiolytic oxidant,^3,13–17^ groundwater bicarbonate concentrations,^18–20^ and redox conditions.^5,21–23^ Radionuclide release into the local environment *via* groundwater requires damage to the storage canister in order for groundwater to contact the spent fuel. Therefore, the role of Fe^II^ ions generated by canister corrosion on spent fuel dissolution have also been studied,^24–26^ showing that Fe^II^ and its corrosion products react with H_2_O_2_ in solution reducing the dissolution of spent fuel. Under geological disposal conditions, H_2_ will be generated by radiolysis and by the anoxic oxidation of canister materials, and the effect of H_2_ on spent fuel dissolution has been shown to have a suppressive effect on dissolution under various conditions.^21,27–31^ Differences in dissolution from SIMFUEL and pure UO_2_ have also been observed due to a greater fraction of H_2_O_2_ dissociation on SIMFUEL caused by differences in surface redox activity.^6^ Epsilon particles of spent fuel have also been shown to affect dissolution by acting as catalytic sites for H_2_ oxidation, as well as the reaction of H_2_ with H_2_O_2_.^24,31^

An important variable that has not yet been sufficiently investigated is the effect of temperature. Temperatures in a repository are expected to be <100 °C throughout the storage lifetime, and will decrease over time as the decay heat generated from radionuclides decreases. There are multiple variables that will determine the temperature profile of a repository with time, including spent fuel burn up, thermal conductivity of bedrock, as well as buffer layer material and thickness to name a few. Recent studies have shown that the temperature at the waste canister surface may be ∼20–30 °C after 10 000 years of storage.^32,33^ Therefore, depending on the storage conditions and the time of storage container failure, temperatures in the repository will vary. Studies on the temperature effect on spent fuel dissolution under repository conditions have shown that an increase in temperature reduces dissolution under a H_2_ atmosphere due to increased uranyl reduction,^34^ whilst dissolution increases with temperature in O_2_ atmospheres.^35,36^ The reported activation energy range for spent fuel dissolution gives values between 15–80 kJ mol^−1^ for the overall oxidative dissolution process.^35–39^ However, there is still a lack of knowledge regarding the temperature effect on the mechanism of H_2_O_2_ decomposition on UO_2_.

With the aim of being able to accurately predict the dissolution behaviour of UO_2_ at the time of container failure, we have studied U dissolution from UO_2_/sodium bicarbonate (NaHCO_3_) suspensions upon H_2_O_2_ addition at 4 bicarbonate concentrations (0.1, 1, 10 and 50 mM) and 4 temperatures (10, 25, 45, and 60 °C). The kinetics and mechanism of H_2_O_2_ decomposition at the UO_2_ surface was analysed as a function of bicarbonate concentration and temperature by monitoring the decomposition of H_2_O_2_ and dissolution of U.

## Experimental

### Materials

Dissolution experiments were conducted using UO_2_ powder. The UO_2_ powder was prepared by reduction of U_3_O_8_ powder under a 10%H_2_ : Ar atmosphere at 1000 °C for 6 hours. The powder was then stored in a flame-sealed glass vial until use to minimize any oxidation of the surface. The structure of the UO_2_ powder was confirmed by XRD according to the procedure previously described in ref. 12. An average crystallite size of 62 nm was obtained, with a cubic lattice constant of 5.46 Å which is consistent with published data for UO_2_.^40,41^ The specific surface area of the powder was measured using the Brunauer–Emmett–Teller method of Kr gas surface adsorption/desorption with a Micromeritics Tristar II instrument giving a surface area of 0.67 ± 0.05 m^2^ g^−1^.

### Dissolution experiments

The dissolution of UO_2_ by reaction with H_2_O_2_ (Fujifilm Wako Pure Chemical, 30%) in NaHCO_3_ (Alfa Aesar) solution was conducted by monitoring the concentrations of U and H_2_O_2_ over the reaction time. It should be noted that irradiation conditions were simulated by the use of commercial H_2_O_2_ which may affect the dissolution compared to H_2_O_2_ generated by radiolysis under deep geological disposal conditions. Suspensions of UO_2_ powder (50 mg) in NaHCO_3_ (70 ml) at different concentrations (0.1, 1, 10 and 50 mM) were prepared in a reaction cell, and pH and ORP values of solution through the experiments are provided in ESI.† To emulate the anoxic conditions of groundwater, the suspensions were purged with Ar for approximately 18 hours prior to the experiment, and purging was continued during the experiment to ensure absence of O_2_. The stability of the system through the dissolution experiments was confirmed by monitoring dissolved U concentrations over the reaction time without H_2_O_2_ addition under select conditions (Fig. S2†). To initiate the reaction, 300 μM H_2_O_2_ was added to the suspension as this concentration has been shown to be optimal for studying H_2_O_2_ induced dissolution with UO_2_.^11^ The temperature within the cell was controlled with a water coolant system to maintain constant temperatures throughout the dissolution experiments. Dissolution experiments were conducted at 10 °C, 25 °C, 45 °C and 60 °C. At intervals during the reaction, samples (∼2 ml) were extracted from the reaction cell and filtered through a 0.45 μm filter to stop the reaction. The filtrate was then tested for U and H_2_O_2_ concentrations as described below. The error in the dissolution experiment methodology was analysed by taking the standard deviation of the calculated H_2_O_2_ pseudo-first order decay constants for H_2_O_2_ decomposition for a dissolution experiment done in triplicate, giving an estimated error of <10%.

### Analytical techniques

U concentrations were measured by ICP-OES with a PerkinElmer Avio-200 spectrometer, where calibration was conducted using appropriate U standards. Measurements were done in triplicate with standard deviations typically being <2% of the measured values.

To measure H_2_O_2_ concentrations, the Ghormley triiodide method was used where H_2_O_2_ reacts with the iodide ion (I^−^) which is converted to the triiodide ion (I_3_^−^) using ammonium heptamolybdate ((NH_4_)_6_Mo_7_O_24_) and the acidic buffer potassium hydrogen phthalate (KHC_8_H_4_O_4_).^42,43^ The absorbance peak of I_3_^−^ at 350 nm was measured using a Shimadzu UV-3600 Plus UV-Vis-NIR spectrophotometer to determine the concentration of H_2_O_2_.

## Results and discussion

### H_2_O_2_ stability in bicarbonate solution

**Fig. 1:**
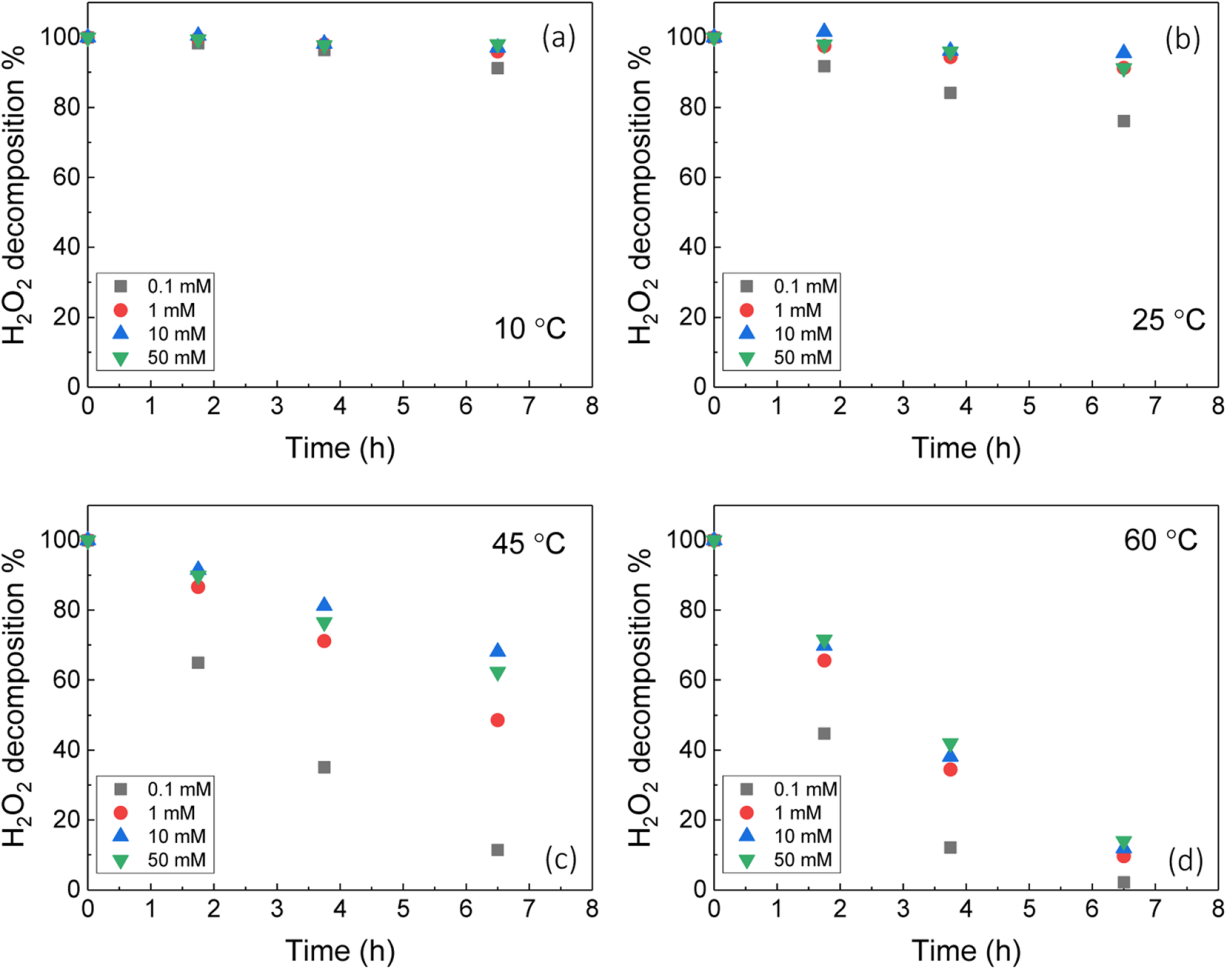
The stability of H_2_O_2_ in bicarbonate solution as a function of temperature (initial concentration 300 μM H_2_O_2_).

To investigate the decomposition of H_2_O_2_ at the UO_2_ surface, it is first necessary to assess the stability of H_2_O_2_ in bicarbonate solution with temperature without UO_2_ as shown in Fig. 1. At concentrations ≥1 mM there was little effect of the bicarbonate concentration on H_2_O_2_ decomposition. However, at 0.1 mM the decomposition occurred more quickly at each temperature. This suggests complex formation between the H_2_O_2_ and bicarbonate in solution which inhibited H_2_O_2_ decomposition. In 0.1 mM bicarbonate, the concentration was lower than the added H_2_O_2_ (300 μM) meaning any stabilisation effect from complexation was lost and H_2_O_2_ decomposed at a faster rate. The H_2_O_2_ decomposition increased with temperature as expected. Further discussions about the kinetics of H_2_O_2_ decomposition on UO_2_ include a background correction for the stability of H_2_O_2_ in bicarbonate solution with temperature.

### U dissolution

**Fig. 2:**
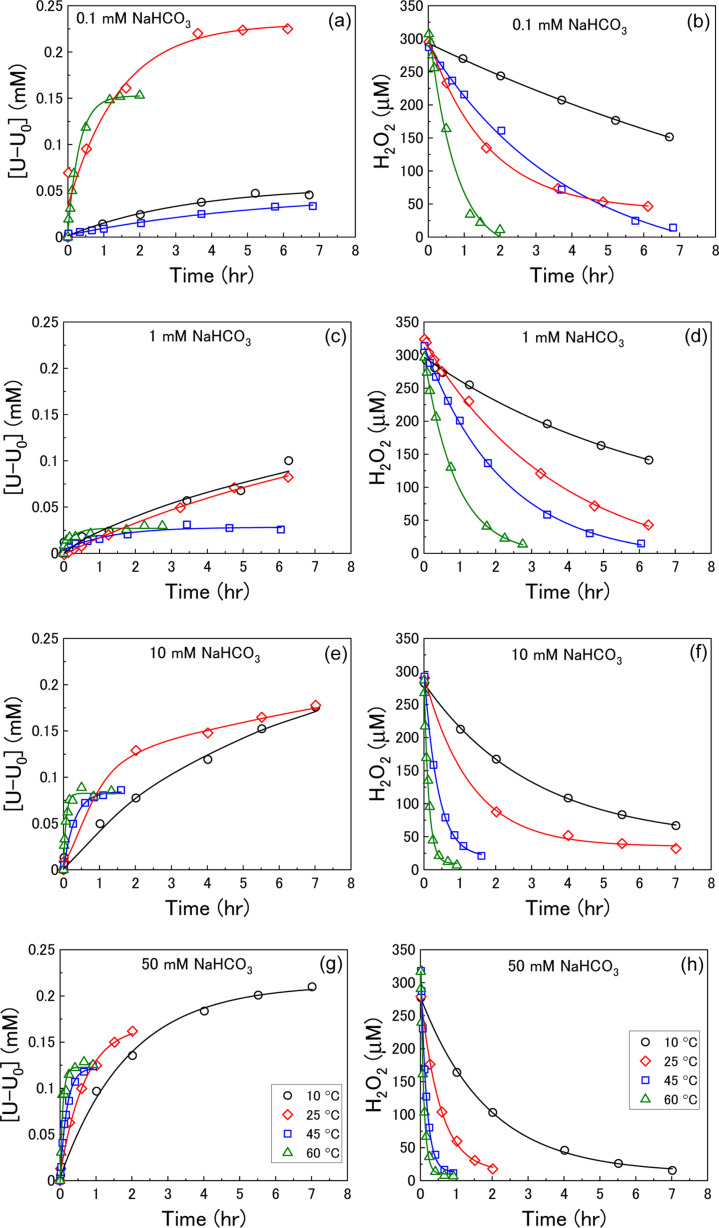
The dissolution of U as a function of temperature in (a) 0.1 mM, (c) 1 mM, (e) 10 mM and (g) 50 mM bicarbonate solution after addition of 300 μM H_2_O_2_, and the corresponding concentration of H_2_O_2_ in (b) 0.1 mM, (d) 1 mM, (f) 10 mM and (h) 50 mM bicarbonate solution (black – 10 °C, red – 25 °C, blue – 45 °C, green – 60 °C).

U dissolution from UO_2_ as a function of temperature was investigated by measuring the concentration of dissolved U over the reaction time after addition of H_2_O_2_ at various bicarbonate concentrations. The measured U dissolution is shown in Fig. 2(a), (c), (e) and (g), where dissolution is given by the measured dissolved U minus the concentration of dissolved U prior to H_2_O_2_ addition (U_0_). The reaction temperature had a significant effect on U dissolution from UO_2_ at each bicarbonate concentration.

At bicarbonate concentrations ≥1 mM, correlations between temperature and dissolution were observed indicating that dissolution is controlled by the formation of U-bicarbonate surface complexes. The first observation is that the initial rate of U dissolution increased with temperature at each bicarbonate concentration. The dissolution rate increase with temperature can be attributed to increased collisions of H_2_O_2_ with the surface leading to higher numbers of oxidative dissolution reactions and subsequent U dissolution. The dissolution rate also increased with bicarbonate at each temperature due to favourable complexation with bicarbonate as described in eqn (6)–(8). The second observation is that the U concentration decreased with increasing temperature. At 45 °C and 60 °C, the dissolved U concentration become constant and the stable value of U could be observed. At 10 °C and 25 °C, the dissolution was slower and the H_2_O_2_ decomposition experiment finished before the stable value of U could be obtained. However, extrapolation of these dissolution profiles indicated that U decreased with increasing temperature. At 0.1 mM bicarbonate, no obvious relationship between temperature and U dissolution was observed. This means that the concentration of bicarbonate is sufficiently low that U-bicarbonate species at the surface do not control dissolution. As the concentration of U exceeds that of bicarbonate, the dissolved uranium is likely in the hydroxide form ((UO_2_)_*m*_(OH)_*n*_^(2*m*−*n*)+^) or a mixture of hydroxide and bicarbonate (*i.e.* (UO_2_)_2_CO_3_(OH)_3_^−^).

### Equilibrium calculation for the U^VI^/bicarbonate system

**Fig. 3:**
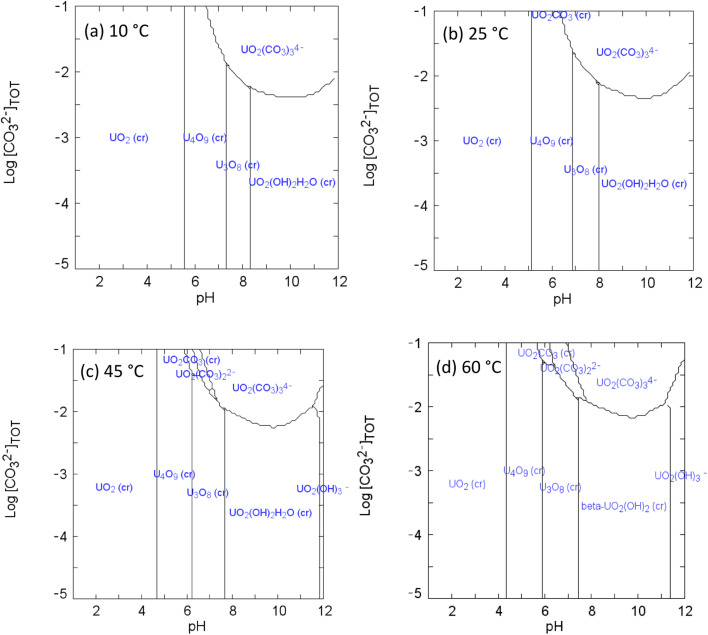
Speciation diagrams of U^VI^ and bicarbonate at (a) 10 °C (b) 25 °C (c) 45 °C (d) 60 °C: [U^VI^]_tot_ = 1 × 10^−3^ M; *I* = 1 × 10^−3^; *E*_h_ = +150 mV.

To investigate the species distribution in the U^VI^/bicarbonate system, thermodynamic calculations were conducted at each experimental temperature. Stability constants were derived using the DQUANT equation derived by Helgeson^44^ which assumes that the temperature dependence of the heat capacity of a dissociation reaction and the temperature dependence of the electrostatic contribution are proportional:6
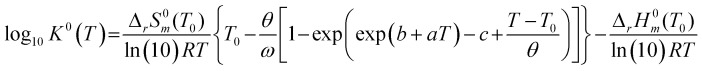
Here, Δ_*r*_*S*^0^_*m*_(*T*_0_) and Δ_*r*_*H*^0^_*m*_(*T*_0_) are the molar entropy and enthalpy of the dissociation reaction at *T*_0_; *R* is the molar gas constant; *θ* = 219 K; *a* = 0.01875 K^−1^; *b* = −12.741; *c* = exp(*b* + *aT*_0_) = 7.84 × 10^−4^; *ω* = 1 + *ac*θ = 1.00322; *T*_0_ = 298.15 K. Thermodynamic data for Δ_*r*_*S*_*m*_(*T*_0_) and Δ_*r*_*H*_*m*_(*T*_0_) taken from the literature^45–47^ were used to calculate the equilibrium constants at 10 °C, 25 °C, 45 °C and 60 °C, and the reactions included in the calculations are provided in ESI.† It should be noted that the calculations do not include the interaction between UO_2_^2+^ and H_2_O_2_ due to a lack of thermodynamic data, and so ternary UO_2_^2+^/CO_3_^2−^/H_2_O_2_ complexes and uranyl peroxides are not considered. The calculated species distribution of U^VI^ are shown in Fig. 3. At 0.1 mM bicarbonate (where the solution pH was 8) a calculated change in speciation from U_3_O_8_ to UO_2_(OH)_2_H_2_O to β-UO_2_(OH)_2_ was found with temperature increase which would explain the lack of order in the U dissolution shown in Fig. 2(a). For the concentrated bicarbonate solutions where the pH was 9.5, the calculated species was UO_2_(CO_3_)_3_^4−^ at all temperatures indicating that carbonate controlled the dissolution of U.

### H_2_O_2_ decomposition kinetics and mechanism

The dissolution of U was induced by the addition of H_2_O_2_ to the UO_2_/bicarbonate suspension. Therefore, the decomposition of H_2_O_2_ over the course of the reaction was studied to investigate the observed dissolution behaviour. The decomposition of H_2_O_2_ over time as a function of temperature for different bicarbonate concentrations is shown in Fig. 2(b), (d), (f) and (h). A clear effect of temperature on H_2_O_2_ was found, where the rate of H_2_O_2_ decomposition increased with temperature at all concentrations of bicarbonate.

To further investigate decomposition of H_2_O_2_ at the UO_2_ surface, the kinetics of decomposition were analysed. Decomposition in the presence of uranium oxides has been shown to follow pseudo-first order kinetics defined by the rate equation:^48^7
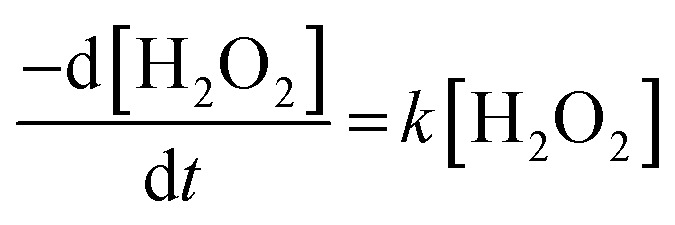


**Fig. 4:**
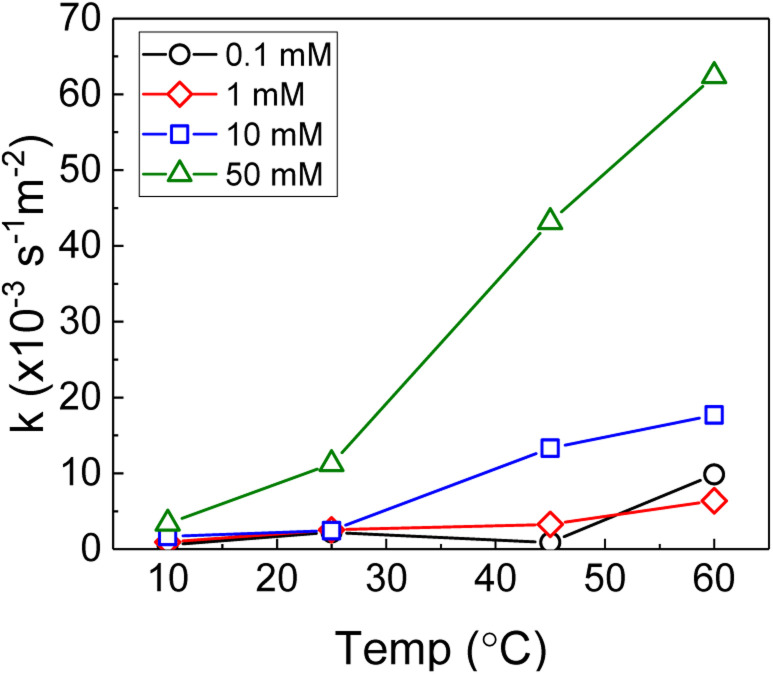
The background corrected pseudo-first order rate constants for H_2_O_2_ decomposition on UO_2_ as a function of temperature in 0.1 mM, 1 mM, 10 mM and 50 mM bicarbonate solution.

Therefore, the pseudo-first order rate constant, *k*, can be obtained from the gradient of a plot of ln[H_2_O_2_] *vs.* time (plots are provided in supplementary information†). The calculated values of *k* were in the range 0.5 to 63 × 10^−3^ s^−1^ m^−2^. A previous study by the authors^12^ investigating U dissolution from U_3_O_8_ in bicarbonate solution at 25 °C showed measured *k* values between 0.7 and 2.7 × 10^−4^ s^−1^ m^−2^, indicating that H_2_O_2_ decomposition is slower on more oxidised forms of U. This effect is likely to be due to the abundance of U^IV^ in UO_2_ relative to U_3_O_8_ facilitating the oxidative decomposition of H_2_O_2_ at the surface *via*eqn (4) and (5). The calculated pseudo-first order rate constants are plotted in Fig. 4 as a function of bicarbonate at different temperatures. The value of *k* increased with temperature for each bicarbonate concentration.

**Fig. 5:**
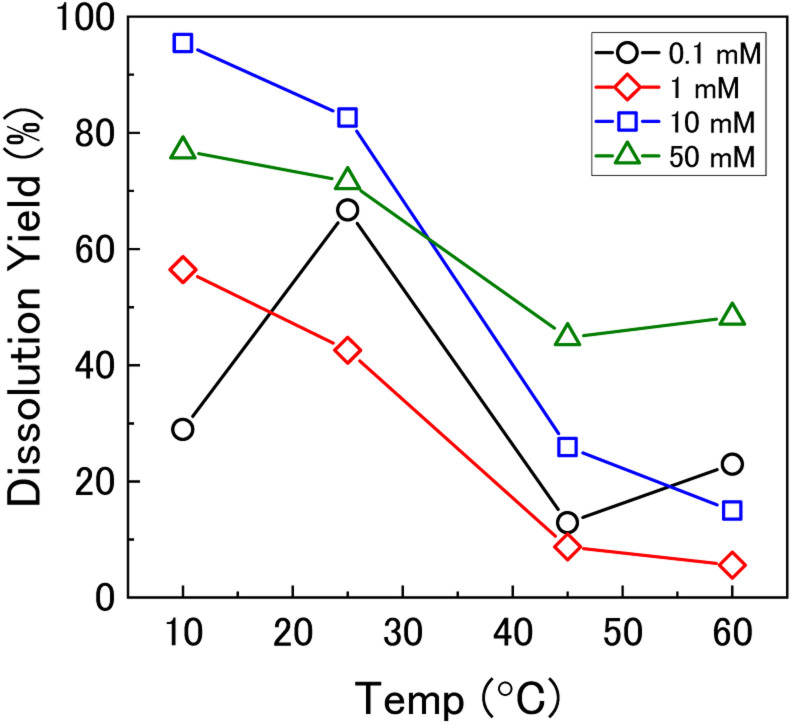
The dissolution yield *vs.* temperature in 0.1 mM, 1 mM, 10 mM and 50 mM bicarbonate solution.

To investigate the mechanism of H_2_O_2_ decomposition further, the contribution of catalytic and oxidative decomposition can be analysed using the dissolution yield. The dissolution yield is defined as the amount of U^VI^ dissolved from the UO_2_ surface per H_2_O_2_ decomposition event at the surface during the reaction, and provides a convenient method to analyse the ratio of catalytic and oxidative decomposition under differing experimental conditions. The dissolution yield was calculated from the final yield at the end of each dissolution experiment as the system was stable at this point (Fig. S6†). The calculated dissolution yields are shown in Fig. 5. At bicarbonate concentrations ≥1 mM, an obvious decrease in the dissolution yield can be seen as temperature increased. This decrease indicated that the H_2_O_2_ decomposition mechanism transitions from oxidative decomposition to catalytic decomposition with temperature. The irregularity of the dissolution yield at 0.1 mM bicarbonate shows that the ratio of oxidative to catalytic decomposition is affected by temperature but has no clear relationship.

### Arrhenius plots for H_2_O_2_ decomposition

The dependence of the rate constant on temperature generally follows the Arrhenius equation:8
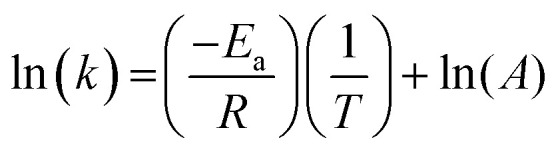


**Fig. 6:**
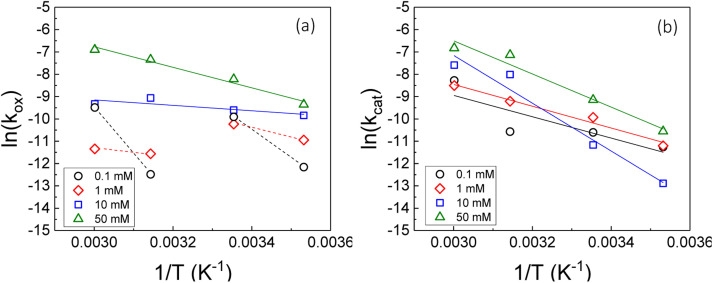
The natural logarithm of the pseudo-first order rate constant for (a) oxidative (*k*_ox_) and (b) catalytic (*k*_cat_) decomposition of H_2_O_2_ on UO_2_ as a function of the inverse of the temperature at various bicarbonate concentrations.

Therefore, the gradient of a ln(*k*) *vs.* 1/*T* plot yields the activation energy for a reaction, *E*_a_. From Fig. 5, the oxidative and catalytic contributions to H_2_O_2_ decomposition could be obtained (*i.e.* a dissolution yield of 60% indicates 60% oxidative and 40% catalytic H_2_O_2_ decomposition). Using this ratio, the values of the pseudo-first order rate constants for oxidative, *k*_ox_, and catalytic, *k*_cat_, decomposition were calculated from the overall rate constant, *k*, shown in Fig. 4. The Arrhenius plots for *k*_ox_ and *k*_cat_ are shown in Fig. 6 and the corresponding values of *E*_a_ are provided in Table 1.

**Table 1:** The calculated activation energies for oxidative and catalytic decomposition of H_2_O_2_ on UO_2_ at different bicarbonate concentrations

NaHCO_3_ (mM)	Oxidative decomposition *E*_a_ (kJ mol^−1^)	Catalytic decomposition *E*_a_ (kJ mol^−1^)
0.1	—	40 ± 5.5
1	—	41 ± 1.6
10	10 ± 1.0	89 ± 5.6
50	38 ± 1.9	62 ± 3.9

For oxidative decomposition of H_2_O_2_ on UO_2_ in 10 mM and 50 mM solution, Arrhenius behaviour was observed. Yet, in ≤1 mM bicarbonate solution, the reaction did not follow Arrhenius behaviour. This indicated a significant effect of temperature on the UO_2_ surface chemistry in ≤1 mM bicarbonate solution, and the resulting H_2_O_2_ decomposition mechanism. For catalytic decomposition, Arrhenius behaviour was observed at all concentrations of bicarbonate. Therefore, any changes to the surface caused by temperature did not significantly affect the catalytic reaction mechanism, and the decomposition of H_2_O_2_*via*eqn (1)–(3) is dependent on the probability of H_2_O_2_ colliding with surface U rather than the form of U. This suggests that catalysis by UO_2_ (U^IV^) and U-bicarbonate (U^VI^) may occur *via* the same mechanism as the surface chemistry change that affected the oxidative decomposition mechanism did not affect the catalytic mechanism. The measured values of *E*_a_ are comparable with the literature data for the overall dissolution reaction as discussed above. A study by de Pablo *et al.*^49^ focused on the individual surface reactions and found *E*_a_ values for UO_2_ oxidation between 30–80 kJ mol^−1^. To the author's knowledge, the *E*_a_ values provided are the first for the oxidative and catalytic H_2_O_2_ decomposition reactions on UO_2_ in simulated geological disposal conditions. The larger *E*_a_ values for catalytic decomposition of H_2_O_2_ at the UO_2_ surface suggests that the reaction of the surface adsorbed hydroxyl radical with H_2_O_2_ requires more energy than the oxidation of U^VI^ to U^V^.

### Effect of temperature on U dissolution

**Fig. 7:**
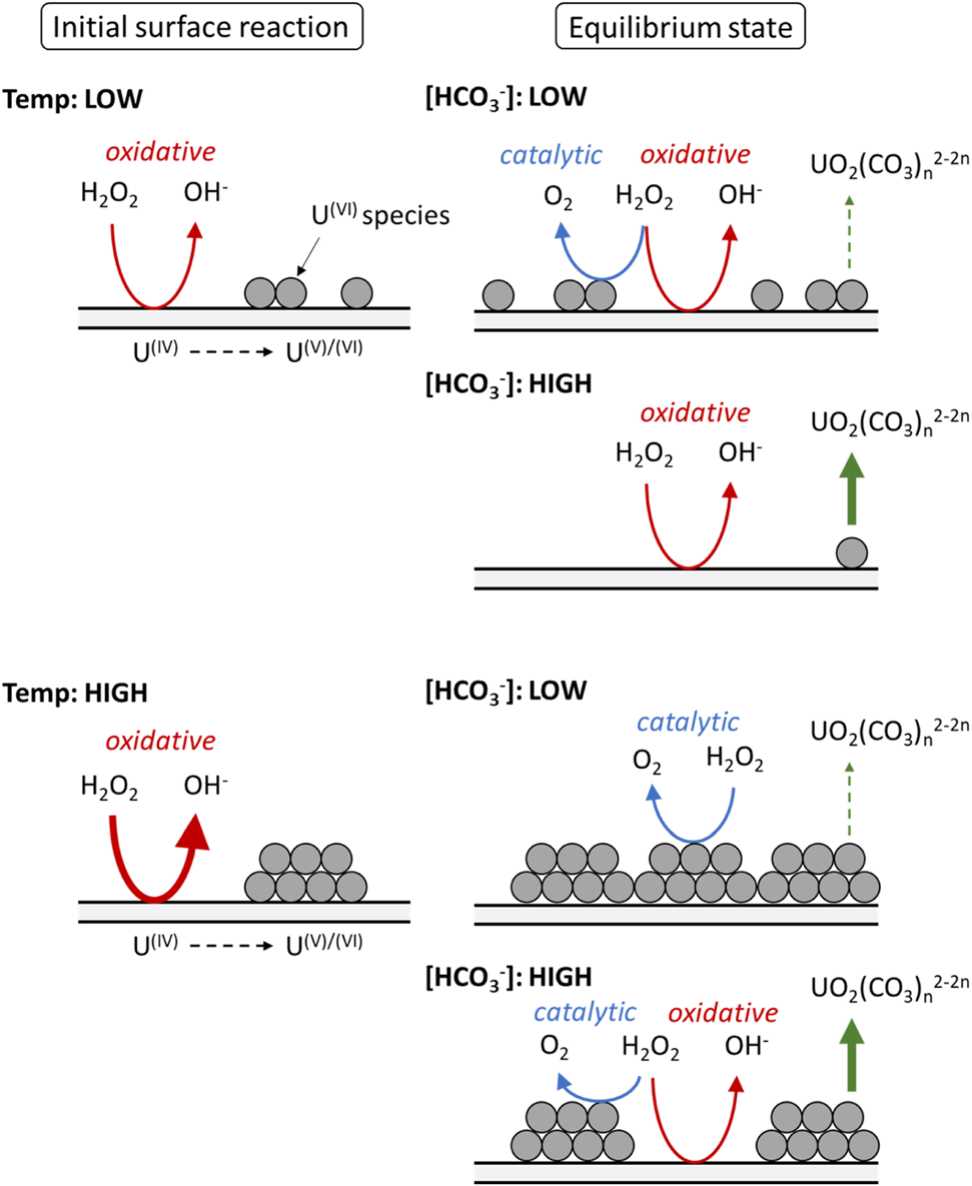
The proposed effect of temperature on the decomposition of H_2_O_2_ at the UO_2_ surface in bicarbonate solution.

Based on the calculated dissolution yields, a pathway for U dissolution from UO_2_ in bicarbonate solution as a function of temperature can be proposed, and is summarised in Fig. 7. Upon addition of H_2_O_2_, the initial oxidative decomposition of H_2_O_2_ occurs on the bare UO_2_, and U^VI^ is generated on the surface which complexes with bicarbonate from solution. These U^VI^ species are expected to be in equilibrium with soluble UO_2_(CO_3_)_*n*_^2−2*n*^, with continuous dissolution of surface U^VI^ species and reprecipitation leading to a transient oxide surface. Otherwise, the formation of a stable surface layer would protect the underlying UO_2_ from oxidative decomposition of H_2_O_2_, and dissolution of U would be inhibited. Raman analysis of the surface oxide after the dissolution experiments showed no alteration to the surface, further indicating a transient oxide surface.

At low temperature, the rate of the initial oxidative decomposition is low, and so the formation of U^VI^ species at the surface proceeds slowly. At low bicarbonate concentrations, the dissolution of the U^VI^ species into solution is also slow, and so the U^VI^ species at the surface partially block the underlying U^IV^ surface. This enables H_2_O_2_ decomposition *via* both catalytic and oxidative pathways. When the bicarbonate concentration is high, the rate of U^VI^ dissolution is high and the surface of the UO_2_ is exposed to oxidative H_2_O_2_ decomposition, and the dissolution yield increases.

At high temperature, the initial surface oxidation reaction proceeds faster than at low temperature, which is evidenced by the higher initial rate of U dissolution in Fig. 2. The increase in U^IV^ oxidation leads to the formation of more U^VI^ species and a larger surface coverage. At low bicarbonate concentrations, these species cover the surface of the UO_2_ due to the low rate of U^VI^ dissolution, and the H_2_O_2_ decomposition mechanism is mainly catalytic. This change in the surface composition may explain the non-Arrhenius behaviour at low bicarbonate concentration shown in Fig. 6, as oxidative decomposition at the UO_2_ surface at higher temperature becomes restricted. With an increase in bicarbonate concentration, the dissolution rate increases leaving parts of the UO_2_ surface exposed, and both catalytic and oxidative H_2_O_2_ decomposition proceeds.

## Conclusion

The decomposition of H_2_O_2_ at the UO_2_ surface in bicarbonate solution as a function of temperature has been investigated, and a pathway to explain the temperature effect has been proposed. U dissolution was controlled by surface U-bicarbonate species. The initial rate of dissolution increased with temperature due to increased collisions of H_2_O_2_ with the surface. The concentration of dissolved U showed an inverse relationship with temperature which was attributed to a transition from oxidative to catalytic H_2_O_2_ decomposition at the UO_2_ surface with increasing temperature. This transition was ascribed to an increased rate of U^VI^-bicarbonate formation at the surface, protecting the underlying UO_2_ and reducing the rate of oxidative decomposition of H_2_O_2_. The catalytic decomposition of H_2_O_2_ proceeded seemingly independently of the nature of the U species at the oxide surface. In 0.1 mM bicarbonate solution, a clear relationship between dissolution and temperature was not observed which was attributed to the formation of both bicarbonate and hydroxide surface species in the bicarbonate deficient system.

## Conflicts of interest

There are no conflicts of interest to declare.

## Supplementary Material

RA-013-D2RA08131H-s001
