# Supplementary material for: UO2 dissolution in bicarbonate solution with H2O2: the effect of temperature
Source: RSC Adv. 2023 Sep 22;13(40):28021–9. doi: 10.1039/d2ra08131h (PMC10517105; doi:10.1039/d2ra08131h)
Supplement: RA-013-D2RA08131H-s001 [file RA-013-D2RA08131H-s001.pdf]

Electronic Supplementary Material (ESI) for RSC Advances.
This journal is © The Royal Society of Chemistry 2023

Supplementary Information

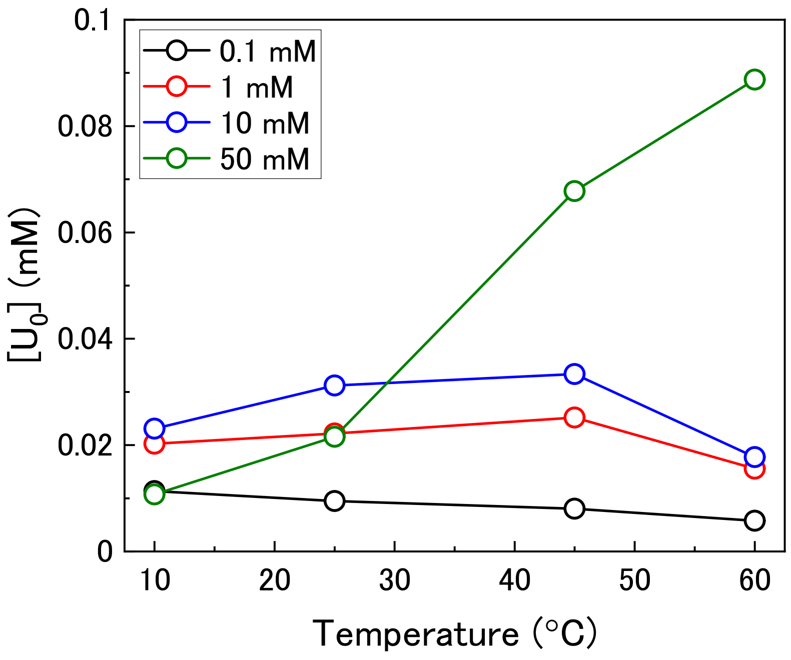

Figure S1: The measured U_0 concentrations at each experimental temperature prior to addition of H_2O_2 for each bicarbonate concentration. The dissolved U concentration at 0.1 mM, 1 mM and 10 mM bicarbonate was relatively unaffected by temperature, whereas in 50 mM bicarbonate the dissolved U concentration increased with temperature. As the UO_2 powder was from the same sample in all tests, the amount of U(VI) is expected to be the same. Therefore, the increasing value of U_0 with 0.1, 1 and 10 mM bicarbonate is logical due to increased complexation of bicarbonate with U(VI), and temperature appears to have a small effect on the complexation. The reason for the change in the temperature effect at 50 mM bicarbonate is unclear, but it may be caused by a shift in the exothermic reaction below:

$$HCO_3^- + H_2O \leftrightarrow H_2CO_3 + OH^-$$

The increased temperature will shift the equilibrium to HCO_3^- which will favour complexation with U(VI) leading to a shift in the equilibrium between dissolved U and deposited U. This effect may not be prominent at lower bicarbonate concentrations.

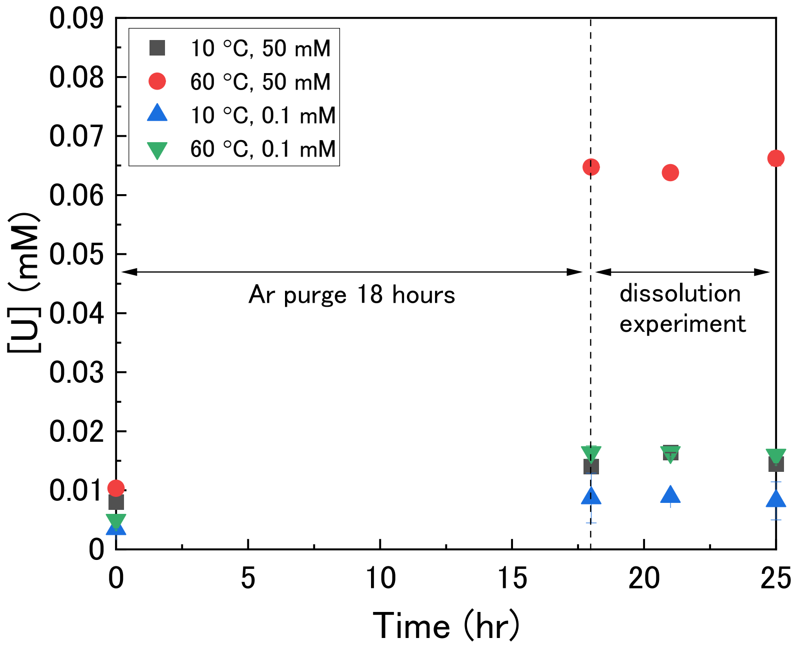

Figure S2: Dissolved U concentrations during preparation and over the course of the dissolution experiment times at 10 and 60 °C in 0.1 and 50 mM $NaHCO_3$ solution. The background concentration of U was stable over the course of the reaction.

Equilibrium constants for the following reactions were calculated and implemented in the thermodynamic calculations. Values for $\Delta_r S_m^0(T_0)$ and $\Delta_r H_m^0(T_0)$ were obtained from ref[1]. For equations marked with * $\Delta_r S_m^0(T_0)$ was calculated from $\Delta_r G_m^0(T_0)$ taken from ref[1] and $\Delta_r H_m^0(T_0)$ taken from ref[2]. For H_2O_2, $\Delta_r S_m^0(T_0)$ was calculated from $\Delta_r G_m^0(T_0)$ taken from ref[3] and $\Delta_r H_m^0(T_0)$ taken from ref[1].

$UO_2^{2+} + H_2O \rightleftharpoons UO_2OH^+ + H^+$

$UO_2^{2+} + 2H_2O \rightleftharpoons UO_2(OH)_2\,(aq) + 2H^+$ *

$UO_2^{2+} + 3H_2O \rightleftharpoons UO_2(OH)_3^- + 3H^+$ *

$2UO_2^{2+} + 2H_2O \rightleftharpoons (UO_2)_2(OH)_2^{2+} + 2H^+$

$3UO_2^{2+} + 5H_2O \rightleftharpoons (UO_2)_3(OH)_5^+ + 5H^+$

$3UO_2^{2+} + 7H_2O \rightleftharpoons (UO_2)_3(OH)_7^- + 7H^+$ *

$UO_2^{2+} + CO_3^{2-} \rightleftharpoons UO_2(CO_3)\,(aq)$

$UO_2^{2+} + 2CO_3^{2-} \rightleftharpoons UO_2(CO_3)_2^{2-}$

$UO_2^{2+} + 3CO_3^{2-} \rightleftharpoons UO_2(CO_3)_3^{4-}$

$2H_2O \rightleftharpoons H_2O_2 + 2H^+ + 2e^-$

$UO_2^{2+} + 2H_2O \rightleftharpoons \beta - UO_2(OH)_2\,(s) + 2H^+$

$UO_2^{2+} + 3H_2O \rightleftharpoons UO_2(OH)_2H_2O\,(s) + 2H^+$

$UO_2^{2+} + CO_3^{2-} \rightleftharpoons UO_2CO_3\,(s)$

$UO_2^{2+} + 0.25H_2O + 1.5e^- \rightleftharpoons UO_{2.25}\,(s) + 0.5H^+$

$UO_2^{2+} + 0.33H_2O + 1.33e^- \rightleftharpoons UO_{2.33}\,(s) + 0.66H^+$

$UO_2^{2+} + 0.66H_2O + 0.66e^- \rightleftharpoons UO_{2.66}\,(s) + 1.33H^+$

$UO_2^{2+} + 2e^- \rightleftharpoons UO_2\,(s)$

Plots of ln[H_2O_2] vs time for the decomposition of H_2O_2 in UO_2/bicarbonate suspensions to determine the pseudo-first order rate constant for H_2O_2 decomposition, pH vs reaction time, and the calculated activation energies, E_a, for oxidative and catalytic H_2O_2 decomposition.

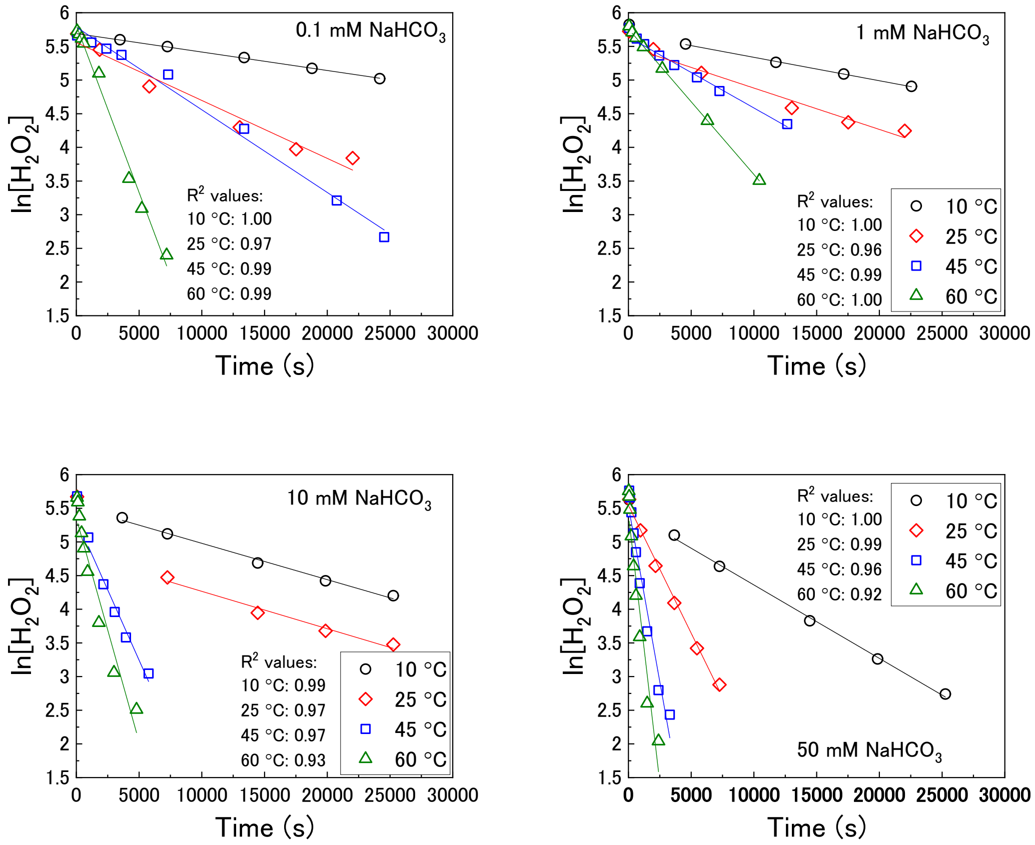

Figure S3: Determination of the pseudo-first order rate constant for H_2O_2 decomposition in UO_2/bicarbonate suspensions.

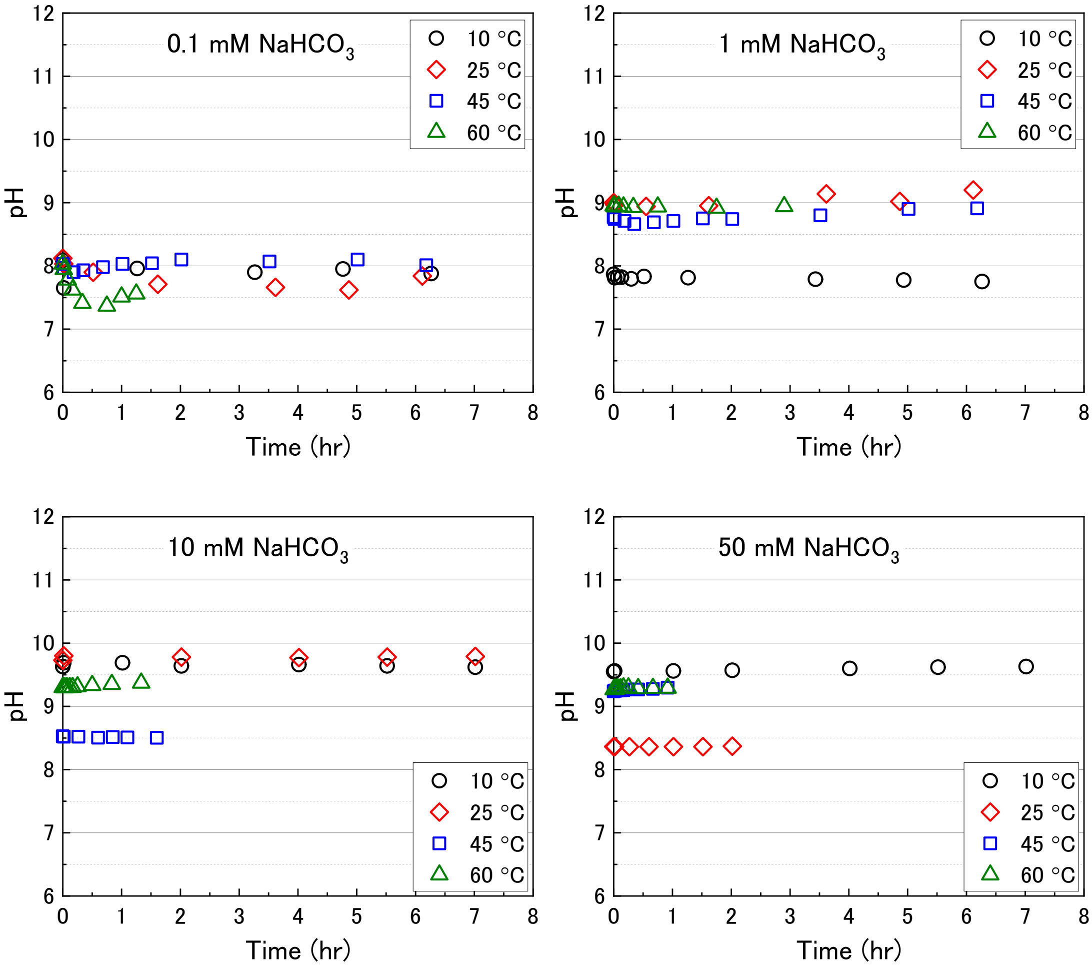

Figure S4: The pH of solution during the dissolution experiments.

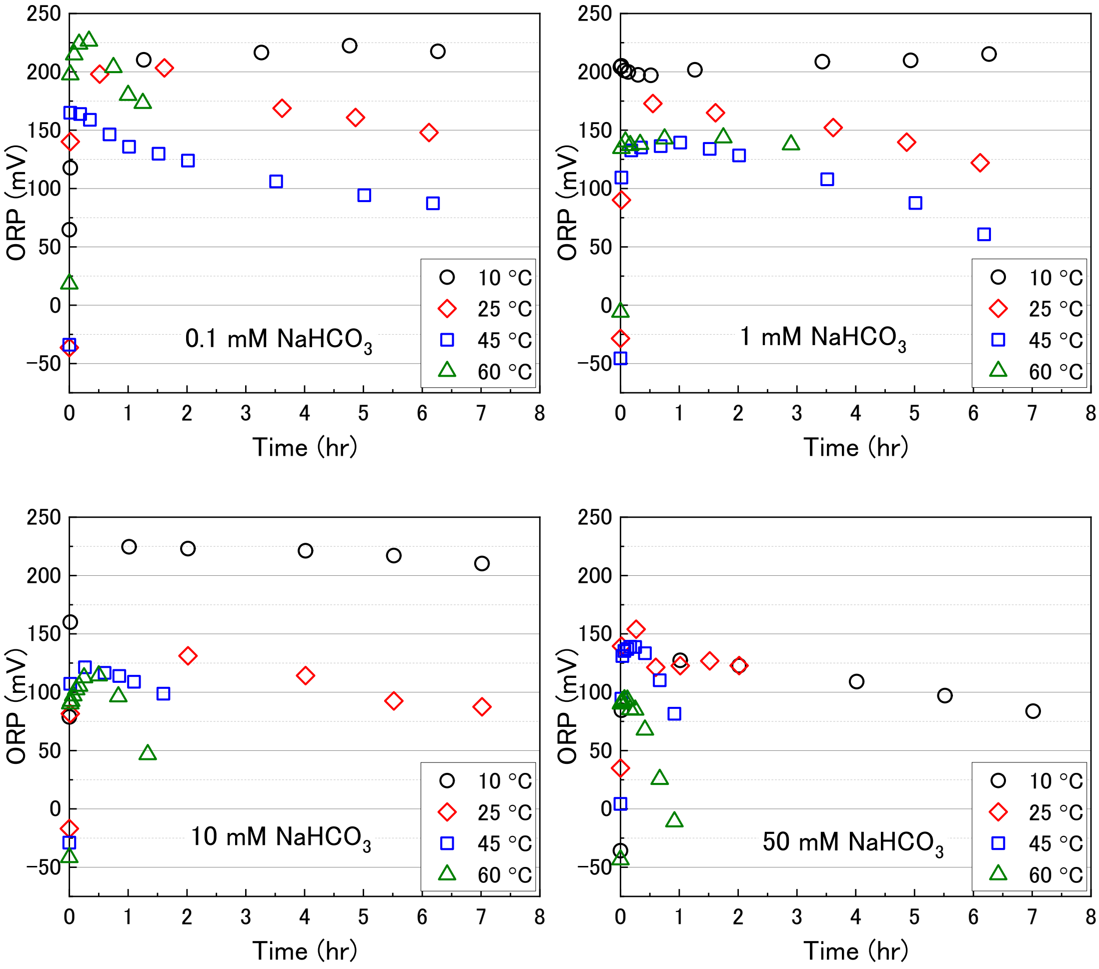

Figure S5: The ORP vs SHE of solution during the dissolution experiments.

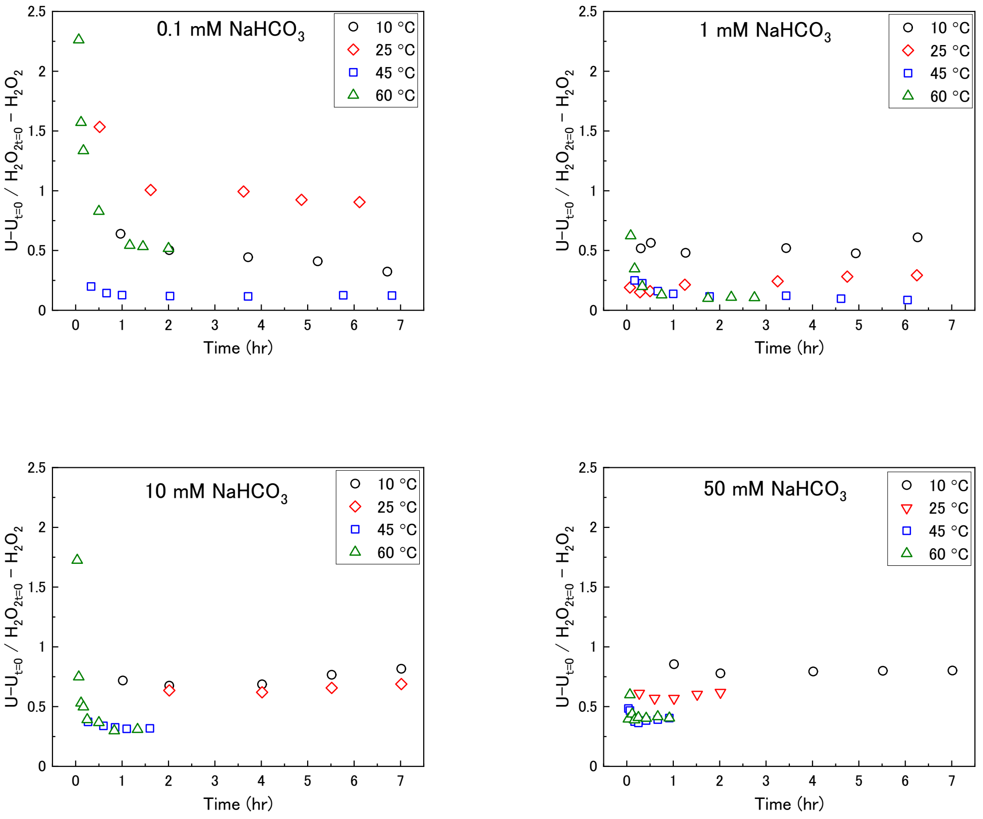

Figure S6: The dissolution yield (U^{t}-$U^{t=0}$ / $H_2O_2^{t=0}$ – $H_2O_2^{t}$) vs time for the dissolution experiments.

References

1. Grenthe, A. V. Plyasunov, W. H. Runde, R. J. Konings, E. E. Moore, X. Gaona, L. Rao, B. Grambow and A. L. Smith, “Second update on the chemical thermodynamics of uranium, neptunium, plutonium, americium and technicium”, 2020, North Holland, Amsterdam, Holland.

2. V. Eliet, I. Grenthe and G. Bidoglio, “Time-resolved laser-induced fluorescence of uranium(VI) hydroxo-complexes at different temperatures,” *App. Spec.*, 2000, **54**, 99-105, doi.org/10.1366/0003702001948.

3. D. D. Wagman, W. H. Evans, V. B. Parker, R. H. Schumm, I. Halow, S. M. Bailey, K. L. Churney and R. L. Nutall, “The NBS tables of chemical thermodynamic properties: selected values for inorganic and C1 and C2 organic substances in SI units,” *J. Phys.*

Chem. Ref., 1982, **11**.
